# Brás Cubas, Quincas Borba, and Rubião: portraits of neuropsychiatry in the novels of Machado de Assis

**DOI:** 10.1055/s-0045-1806828

**Published:** 2025-04-27

**Authors:** Juliana de Castro Vilanova, Antonione Santos Bezerra Pinto, Giuliano da Paz Oliveira

**Affiliations:** 1 Instituto de Ensino Superior do Vale do Parnaíba, Parnaíba PI, Brazil.; 2 Universidade Federal do Delta do Parnaíba, Parnaíba PI, Brazil.

**Keywords:** Neurology, Neurosciences, Neuropsychiatry, Literature, Medicine in Literature

## Abstract

The intersection of literature and neuroscience provides a fascinating way to explore human behavior through fictional narratives. Brazilian literature, particularly the work of Machado de Assis, excels in portraying characters with neuropsychiatric conditions. This work aims to establish connections between the fictional representations of human behavior in Machado's classic works and neurological conditions described by contemporary neuroscience. In *The Posthumous Memoirs of Brás Cubas* and *Quincas Borba* , Machado's characters exhibit behaviors that align with modern neurological diagnoses. For example, Brás Cubas experiences episodes resembling delirium, characterized by mental confusion and altered cognition, while Quincas Borba shows traits of attention deficit hyperactivity disorder (ADHD) and bipolar disorder. Rubião, the protagonist of Quincas Borba, meets certain criteria for dementia, displaying visual hallucinations and cognitive fluctuations. By analyzing Machado's characters through a neuropsychiatric lens, we can appreciate his remarkable ability to depict complex mental conditions, many of which were not fully understood by medicine at the time.

## INTRODUCTION

**Figure 1:**
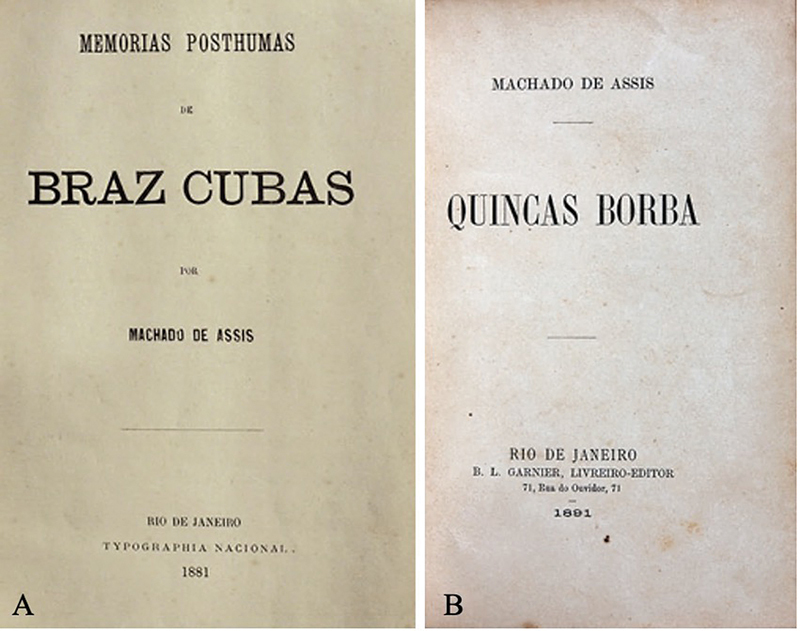
Source: Itaú Brasiliana Collection/Photo reproduction Horst Merkel. ( **A** ) Cover of the book *The Posthumous Memoirs of Brás Cubas.* Rio de Janeiro, 1881. ( **B** ) Cover of the book *Quincas Borba* . Rio de Janeiro, 1891.

Neuroscience explores the biological and neural mechanisms underlying mental and behavioral processes. The intersection of literature and neuroscience explores narratives with diverse human experiences, including neuropsychiatric conditions. While studies have focused on authors such as Cervantes and Shakespeare, little research has been done on Brazilian authors. 1 2 Machado de Assis (1839–1908), one of Brazil's most importantwriters, created characters with behaviors and cognitive patterns interpretable through neuropsychiatry, particularly in his novels *The Posthumous Memoirs of Brás Cubas* (1881) *,* ( Figure 1A ), and *Quincas Borba* (1891) ( Figure 1B ). Machado's own experience with epilepsy, a condition with which he struggled throughout his life, may have contributed to his nuanced portrayal of mental and neurological conditions. 3 He used a unique narrative to explore the cynical and ironic thoughts of Brás Cubas, Rubião, and Quincas Borba, revealing behaviors suggestive of cognitive, mood, and neurodevelopmental disorders. 4 5

Understanding these representations provides insights into mental and neurological conditions, enriching both literature and neuroscience. This research aims to identify clinical features in Machado's works that suggest possible neuropsychiatric diagnoses based on current scientific knowledge.

## MACHADO DE ASSIS AND NEUROPSYCHIATRY

### Brás Cubas

In *The Posthumous Memoirs of Brás Cubas* , the protagonist Brás Cubas experiences an episode consistent with the *Diagnostic and Statistical Manual of Mental Disorders* , 5th edition (DSM-V) criteria for *delirium* —an acute confusional state characterized by short-term confusion and changes in cognition. 3 6 7 Among the criteria described in the book are the presence of disturbances in attention and consciousness, with disorganization of thought, which develop over a brief period of time. 4 7 There are also some associated clinical features necessary for diagnosis, such as hallucinations and delusions. 4

To fulfil all the criteria, the clinical history described seems to be a direct physiological complication of another medical condition—the pneumonia that Brás Cubas presented at the time. In the novel, Machado also points out one of the greatest risk factors for delirium, the protagonist's advanced age (64 years old). 4 7 Table 1 summarizes the main criteria and the corresponding excerpts from the book.

**Table 1:** Diagnostic criteria for delirium in the character of Brás Cubas and corresponding passages in *The Posthumous Memoirs of Brás Cuba* *s*

DSM-V criteria	Corresponding passages
A disturbance in attention and awareness.	“Her son felt satisfied, hearing those dignified and forceful words, while I wondered to myself what the sparrowhawks might say of us, if Buffon had been born a sparrowhawk …”
The disturbance develops over a short period of time, represents a change from baseline attention and awareness, and tends to fluctuate in severity during the course of a day.	“My delirium was beginning. [...]what went on in my head during some twenty or thirty minutes.”
An additional disturbance in cognition.	“Finally, restored to human form, I saw a hippopotamus come up and carry me off.”
There is evidence from the history, physical examination, or laboratory findings that the disturbance is a direct physiological consequence of another medical condition, substance intoxication or withdrawal, or exposure to a toxin, or is due to multiple etiologies.	“I died of pneumonia...”
The disturbances are not explained by another preexisting, established, or evolving neurocognitive disorder and do not occur in the context of a severely reduced level of arousal.	−

Abbreviation: DSM-V, Diagnostic and Statistical Manual of Mental Disorders, 5th edition.

The term *delirium* was formalized by Celsus in the 1st century AD but remained ambiguous until the 19th century, being used to describe both general madness and acute changes associated with fever. Before its systematic inclusion in the DSM-III in 1980, the term was used to refer to more than 30 conditions, such as metabolic encephalopathy and toxic psychosis. In this context, the author anticipated the modern description of this condition in his works, accurately describing features that would later be formally delineated by medical science. 8

The presence of micropsia, macropsia, dysmorphopsia, and the quick-motion phenomenon strongly suggests Alice in Wonderland Syndrome (AIWS)—a perceptual disorder whose name refers to the book *Alice's Adventures in Wonderland* , by Lewis Carroll (1832–1898)—a contemporary of Machado de Assis, although AIWS was described in 1955 by the psychiatrist John Todd. This syndrome involves distortions in visual perception, body image, and the experience of time. One study found that infectious diseases were the second most common cause, accounting for ∼ 22.9% of cases. 9 10

A recent study used functional magnetic resonance imaging to map lesion sites and analyze their connectivity to other brain regions. The results showed that although AIWS lesions occurred in many different parts of the brain, they followed a specific pattern of connectivity. This network was linked to the right extra-striatal body area, which is activated by visualizing body parts, and the inferior parietal cortex, which is involved in processing size and scale. This pattern was unique to AIWS compared with lesions causing other neuropsychiatric disorders. 9

### Quincas Borba

The enigmatic Quincas Borba presents traces of attention deficit hyperactivity disorder (ADHD) during his youth. This condition is characterized by attention deficit and motor hyperactivity, leading to significant impairment in academic/occupational, family and/or social functioning. 4 11 The author describes the character's impulsivity and hyperactivity, which began around the age of 9—in accordance with the DSM-V criterium that requires the onset of symptoms before 12 years old. 4 6 Additionally, ADHD symptoms must occur in multiple environments, as described in the book. 4 7 Thus, although the literary description strongly suggests a diagnosis of ADHD with a hyperactive presentation in the character, the evidence is insufficient to fully meet the diagnostic criteria, particularly regarding inattention and social or academic impairment. 7

In a historical context, the physician Alexander Crichton was a pioneer when, in 1798, he described the characteristics we now associate with ADHD and referred to them as “pathological inattention.” However, the modern concept of the disorder was not solidified until the publication of the DSM-III. Thus, although Machado did not anticipate the first description of the disorder, the features present in his work are consistent with concepts that would be formally described years later. 12

Additionally, Quincas Borba presents signs of bipolar disorder (BD) in his adult life, with frequent mood fluctuations and behavior changes. 5 7 Especially, there is evidence of manic episodes, marked by inflated self-esteem, grandiose delusions, and psychotic features, suggesting BD type I. 5 7 13 The character also exhibits accelerated, disorganized thinking, with rapid changes of subject and difficulty in drawing conclusions, characteristic of the flight of ideas—a typical symptom of the manic episode. 5

Throughout the books, there are multiple descriptions that fulfill another diagnostic criterion for a manic episode: an increase in goal-directed activity. This can be observed in the context of his Humanitas philosophy, which is a dominant theme in most of his speeches and is also the subject of four books he has written. 4 Unfortunately, there is no description of the duration of the episodes of altered mood to complete the DSM-V criteria ( Table 2 ), but the narrative suggests that these episodes were long-lasting, reinforcing the hypothesis of a diagnosis of BD. 7

**Table 2:** Diagnostic criteria for Quincas Borba's manic episode and corresponding passages in *Quincas Borba*

DSM-5 criteria	Corresponding passages
A distinct period of abnormally and persistently elevated, expansive, or irritable mood and abnormally and persistently increased activity or energy, lasting at least one week and present most of the day, nearly every day (or any duration if hospitalization is necessary).	“Thus, the quirks, the frequent mood swings, the impulses without motive, and the disproportionate tenderness were nothing more than signs of the total ruin of the brain.”
During the period of mood disturbance and increased energy or activity, three (or more) of the following symptoms are present to a significant degree and represent a noticeable change from usual behavior: - Inflated self-esteem or grandiosity; - Flight of ideas or subjective experience that thoughts are racing; - Increase in goal-directed activity or psychomotor agitation - Decreased need for sleep (e.g., feels rested after only 3 hours of sleep); - More talkative than usual or pressure to keep talking; - Distractibility; - Excessive involvement in activities that have a high potential for painful consequences.	“Because immortality is my lot or my dowry, or whatever better name there is. I will live perpetually in my great book.” “He mixed his own and others' ideas, all sorts of imagery, idyllic, epic, to such an extent that Rubião wondered how a man, who was going to die in a few days, could so gallantly address those matters.” “Some days later, Quincas Borba read to me his great masterwork. It spanned four large manuscript volumes, a hundred pages each, written in a small hand and peppered with Latin citations.” “I put my hand into my vest pocket and failed to find my watch. The final disillusion! Borba had stolen it from me during the embrace.”
The mood disturbance is sufficiently severe to cause marked impairment in social or occupational functioning or to necessitate hospitalization to prevent harm to self or others, or there are psychotic features.	“Quincas Borba was not only mad, but he also knew that he was mad, and this shred of consciousness, like a flickering lamp in the gloom, greatly aggravated the horror of the situation.”
The episode is not attributable to the physiological effects of a substance or to another medical condition.	−

Abbreviation: DSM-V, Diagnostic and Statistical Manual of Mental Disorders, 5th edition.

The nosological concept of BD originated in France with the studies of Jean-Pierre Falret and Jules Baillarger in 1856. The first formal diagnosis was made by Falret, who described “circular insanity” characterized by alternating episodes of mania and depression. Although Machado de Assis did not fully anticipate the medical literature on the disorder, his literary descriptions show remarkable agreement with concepts and diagnostic criteria that would be systematically established only decades later, in the various editions of the DSM. 14

Machado de Assis's description of Quincas Borba is in line with recent studies showing that children with ADHD have a higher risk of developing BD in adulthood, with around 20% of ADHD patients having comorbid BD. 15

## Rubião

Rubião, the main character of *Quincas Borba* , inherits a fortune and begins to live lavishly in Rio de Janeiro. Over time, he begins to exhibit symptoms consistent with a dementia syndrome—possibly Lewy body dementia (LBD), the second most common form of degenerative dementia. Its clinical features were first described in 1976 by psychiatrist Kenji Kosaka, who later proposed the term “Lewy body disease” in 1980. 16 Initially, the character exhibits a series of grandiose delusions, such as the belief that he holds the title of marquis. 5

As the dementia process progressed, Rubião developed visual hallucinations and illusions, which are hallmarks of LBD. 17 For example, he alters his physical appearance to resemble what he refers to as his younger self—Napoleon III. Toward the end of the book, his perception of reality becomes progressively more impaired, and he begins to have increasingly complex, vivid, and detailed hallucinations, including a passage in which he converses with an imaginary empress. 5 Another striking feature that further supports the LBD hypothesis is the cognitive fluctuation associated with the rapid progression of the disease. 5 17

Therefore, the primary diagnostic hypothesis for Rubião is LBD, as he meets the criteria for probable LBD, with progressive cognitive decline and at least two key clinical features: fluctuating cognition and visual hallucinations. In addition, the text describes clinical evidence that may support this diagnosis, including the presence of delusions and postural instability, as suggested by his use of a cane throughout the narrative. 17 In this way, Machado de Assis's work depicts clinical features that would be formally grouped under the diagnosis of LBD decades later.

Other possible diagnoses for Rubião include frontotemporal dementia (FTD), a neurodegenerative disorder that affects the frontal and/or temporal lobes. This hypothesis is based on significant changes in his social behavior and personality, with an early onset (around the age of 41). 5 18 The protagonist exhibits disinhibition and perseverative behavior, particularly in his persistent and inappropriate attempts to get closer to Sofia, the wife of his friend. One key example is Rubião's intrusion into Sofia's vehicle, where he makes grand claims and revisits experiences shaped by a deteriorating state of mind. 5 However, the narrative does not fully meet the diagnostic criteria for FTD, which require the presence of at least three features: disinhibition, apathy, loss of sympathy/empathy, perseverative/compulsive behaviors, hyperorality, and dysexecutive neuropsychological profile. 19

Another potential diagnosis is neurosyphilis, characterized by memory and judgement problems, personality changes, and psychiatric symptoms such as depression, mania, or psychosis. This hypothesis is supported by the fact that neurosyphilis was a leading cause of dementia in the preantibiotic era, when the book was written. 3 20

In conclusion, an analysis of Machado de Assis' characters through a neuropsychiatric lens highlights his ability to depict psychological conditions long before they were understood by medicine. Machado not only anticipated some medical descriptions decades ahead but also captured concepts aligning with modern diagnostic criteria, reflecting his exceptional literary and observational insight into psychological complexity.
